# The Effect of Albuterol Spray on Hypoxia and Bronchospasm in Patients with Chronic Obstructive Pulmonary Disease (COPD) under General Anesthesia: A bouble-Blind Randomized Clinical Trial

**DOI:** 10.4314/ejhs.v33i3.12

**Published:** 2023-05

**Authors:** Seyedmahrokh A Maddah, Akbar Barzegari

**Affiliations:** 1 Department of Anesthesiology, Faculty of Medicine, Golestan University of Medical Sciences, Gorgan, Iran

**Keywords:** Albuterol, Pulmonary Disease, Chronic Obstructive, Bronchial Spasm, Hypoxia

## Abstract

**Background:**

Patients with chronic obstructive pulmonary disease (COPD) experience an increased risk of perioperative pulmonary complications. The aim of this study was to evaluate the effect of albuterol spray on hypoxia and bronchospasm in patients with COPD under general anesthesia.

**Methods:**

This single-center, double-blind, parallel-group, randomized clinical trial was performed on 120 smoking patients with COPD who were referred to 5 Azar Educational Hospital in Gorgan, Northern Iran, in 2021. Twenty minutes before general anesthesia and also after completion of surgery and before extubation, 60 patients in the intervention group were inhaled with 2 puffs of albuterol spray. In the control group, patients were inhaled with 2 puffs of placebo spray. In perioperative period, the occurrence of wheezing, bronchospasm, coughing, hemodynamic changes, postoperative shivering, dyspnea, and nausea and vomiting were evaluated in all patients. The Consolidated Standards of Reporting Trials (CONSORT) checklist was used to report important aspects of this study.

**Results:**

The mean age of the patients was 52.34 ±8.95 years, and 115 (95.8%) of them were males while the rest were females. The difference between systolic blood pressure before induction of anesthesia (after administration of albuterol spray) between the group receiving albuterol spray and the group not receiving it was statistically significant (p=0.04). Also, the difference between the mean arterial oxygen saturation before tracheal extubation (after re-administration of albuterol spray) between the albuterol spray group and the non-albuterol group was statistically significant (p = 0.03). Wheezing and recurrent cough after induction of anesthesia and after extubation (after albuterol spray administration) was lower in the albuterol group than in the control group (p<0.05). No significant side effects were detected in the albuterol-treated group.

**Conclusion:**

According to the results of this study, it seems that the prophylactic use of albuterol spray is useful in reducing the incidence of wheezing and recurrent cough before induction of anesthesia in COPD patients with smoking.

## Introduction

Chronic obstructive pulmonary disease (COPD) is a common chronic disease, third leading cause of death and increasingly important cause of morbidity and disability in the world. It is characterized by poorly reversible and progressive airflow obstruction (1). Genetic and environmental risk factors such as exposure to tobacco smoke, indoor air pollution and occupational dusts, fumes and chemicals are important risk factors for COPD (2). Among the multiple risk factors, cigarette smoking is the strongest risk factor for COPD. Carbon monoxide in cigarette smoke has 200-300 times greater affinity for hemoglobin than oxygen and produces carboxyhemoglobin, leading to tissue hypoxia (3). Considering that many of COPD patents will need surgery and general anesthesia during their lives, these patients should be evaluated for hypoxia, bronchospasm, and laryngospasm in perioperative period by conducting a thorough clinical history and physical examination and performing pulmonary function testing (PFT) and arterial blood gases (ABG) analysis (4,5).

Patients with COPD pose a challenge to the anesthesiologists because intraoperative and postoperative complications occur more commonly in these patients, which can lead to prolonged hospital stay and increased mortality (6). Therefore, it is important to take proper measures to reduce the incidence of postoperative pulmonary complications to improve the quality of surgical care and reduce the medical cost (6,7).

Inhaled bronchodilators such as albuterol, a short acting β agonist, are widely prescribed for the symptomatic relief in patients with COPD. Beta-2 adrenergic receptor agonists (β2-agonists) can result in bronchodilatation by stimulating the β2-adrenoceptor present on airway smooth muscle and other cells in the airway (8). Albuterol can often decrease symptoms of airflow obstruction by bronchodilatation, decreasing dyspnea, and improving quality of life (9,10).

Previously, it has been shown that using prophylactic salbutamol spray before surgery, reduces the incidence of bronchospasm and coughing in heavy smoking patients under general anesthesia (11). Also, it has been indicated that using salbutamol aerosol decreases the occurrence of postoperative bronchospasm and pulmonary infiltration in patients with mild-to-moderate COPD (12). However, there is limited evidence regarding the efficacy of perioperative inhaled albuterol on hypoxia and bronchospasms in COPD patients who underwent elective surgeries using general anesthesia. Therefore, the aim of this study was to evaluate the effect of albuterol spray on hypoxia and bronchospasm in patients with COPD under general anesthesia. We hypothesized that using perioperative albuterol spray would reduce hypoxia and bronchospasm in patients with COPD under general anesthesia.

## Methods

In a double-blind randomized clinical trial, after obtaining review and obtaining adequate permissions from the University's Ethics Committee and informed consent from patients, 120 patients who were a candidate for elective surgeries under general anesthesia at 5 Azar Educational Hospital in Gorgan, Iran, were recruited, in 2021.

Patients with previously confirmed diagnosis of COPD, class I or II of American Society of Anesthesiologists, aged between 18 and 65 years and had history of cigarette smoking were included. The exclusion criteria were patients' unwillingness to participate or discontinue the study at any time during the study period, prolongation of surgery duration (more than 3 hours), cardiac dysfunction or heart failure, intraoperative blood loss and the need for transfusion, allergy to salbutamol preparations, smoking cigarettes during 12 hours before surgery, exacerbation of COPD, concomitant pulmonary arterial hypertension (defined as a mean pulmonary arterial pressure > 25 mmHg) and hemodynamic changes >20% from the baseline (blood pressure, heart rate and reduction in SpO2) that need to be managed.

Patients who fulfilled the inclusion criteria were randomly allocated using block randomization method to two equally sized groups of A and B (n=60) by a nurse anesthetist. For ensuring allocation concealment, the sequentially numbered, opaque, sealed envelope technique was used by a nurse who was unaware of the study groups.

In the operating room, all patients were connected to electrocardiography, peripheral oxygen saturation (SpO2) and non-invasive blood pressure monitor and all the basal parameters were recorded. All patients were placed under general anesthesia using a similar anesthetic protocol including midazolam (0.02 mg/kg), fentanyl (3-5 µ/kg), lidocaine (1 mg/kg), propofol (2 mg/kg) nesdonal (5 mg/kg) and cisatracurium (0.2 mg/kg) to facilitate endotracheal intubation. After 3 minutes, patients were intubated with appropriate sized cuffed endotracheal tube and administered a mixture of 50% nitrous oxide (N2O), oxygen, and isoflore 1.5 MAC for maintenance of anesthesia. The continuous mandatory ventilation (CMV) mode was initiated after placing the tracheal tube.

Twenty minutes before anesthesia, patients in the group A were administered two puffs of albuterol (2 X 100 micrograms) orally via a metered-dose inhaler, whereas those in the control group (group B) were given a placebo spray in the same way. Also, the shape of the placebo spray was the same as the albuterol spray. After completion of surgery, patients in group A (n=60) received two puffs of albuterol via the endotracheal tube, whereas those in the control group (group B) were given a placebo spray in the same way. At the end of surgery after discontinuation of anesthetic medications, neostigmine 0.04 mg/kg and atropine 0.02 mg/kg were used for muscle relaxant reversal.

**Primary and secondary outcomes measure**: A lung auscultation was performed to assess wheezing, before administration of albuterol spray and 20 minutes later (after endotracheal intubation), by an anesthesiology resident who was blinded to the study groups. The diagnosis of bronchospasm, during anesthesia, was based on the feel of anesthesiologist that the reservoir bag was too tight when squeezing it (tight bag), in addition to significant depressed SpO2 values. During emergence, coughing was observed and recorded by the same resident blinded to the study protocol and the anesthesia regimen. Patients' systolic blood pressure, diastolic blood pressure, mean arterial blood pressure and heart rate were measured and recorded (during surgery every 10 minutes and during 1 hour recovery period every 15 minutes). Also, occurrence of postoperative shivering, dyspnea, and nausea and vomiting were evaluated in patients.

**Sample size**: A priori sample size were calculated using GPower3.1 with the formula for calculation of samples of repeated measures, based on a presumed effect size of 0.2, a statistical power of 80%, and a type I error of 5%. The overall proper sample size was found to be 108 participants. We therefore recruited 120 patients to account for any dropouts.

**Statistical analysis**: After data collection, statistical analysis was performed using descriptive and inferential statistics via Statistical Package for the Social Sciences (SPSS) software (version 18.0, SPSS Inc., Chicago, IL, USA). Shapiro-Wilk test was used to assess normality of the data. Chi-Square/Fisher exact test and t-test were used for qualitative and quantitative variables, respectively. A P-value less than 0.05 was considered statistically significant. The Consolidated Standards of Reporting Trials (CONSORT) checklist was used to report important aspects of this study.

**Ethical considerations**: The present study was carried out in accordance with the principles of the Declaration of Helsinki and after the approval of the Institutional Ethics Committee (code: IR.GOUMS.REC.1400.272). The researchers explained the objectives of the study to the participants, and informed consent was obtained from all participants. Also, the protocol of this study was registered in the Iranian Registry of Clinical Trials Database (IRCT20170108031818N1).

**Data sharing**: All relevant data and methodological details pertaining to this study are available to any interested researchers upon reasonable request to the corresponding author.

## Results

**Figure 1:**
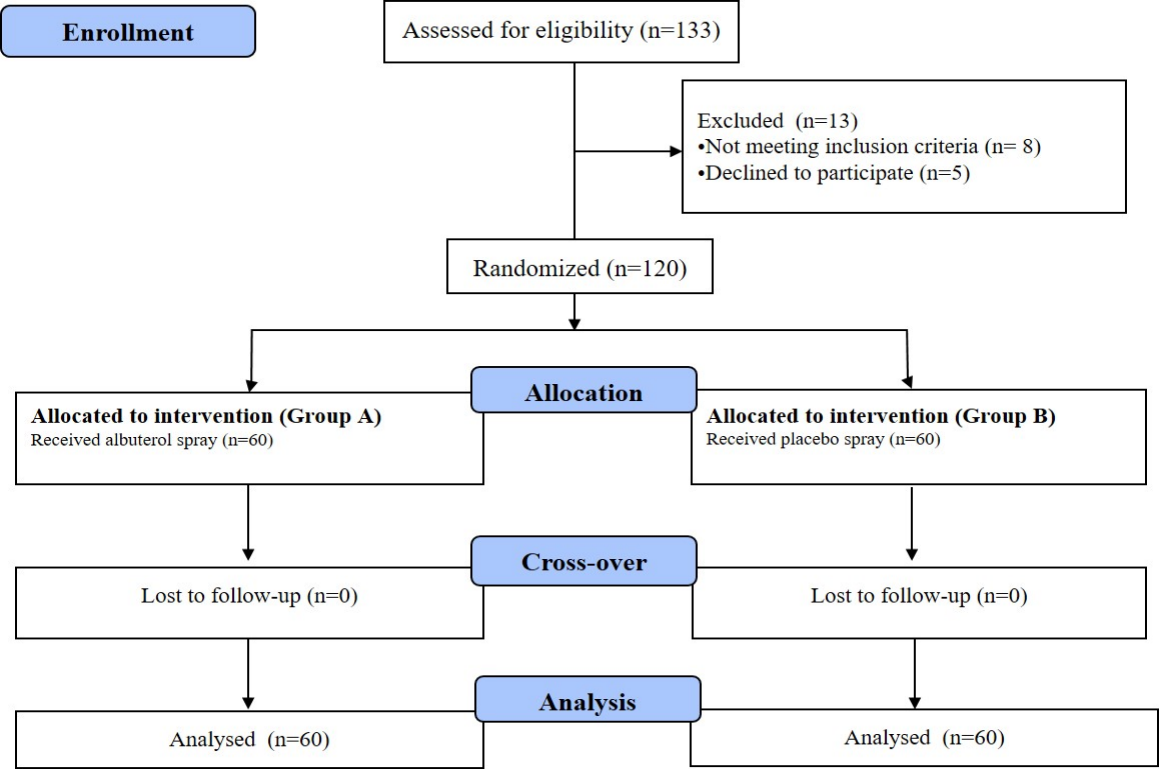
Recruitment and allocation into groups of study subjects

In this study, 133 patients in 5-Azar Hospital in Gorgan were evaluated in terms of inclusion and exclusion criteria. Eight patients did not meet the inclusion criteria while 5 patients did not agree to participate in the study. One hundred twenty patients were randomly assigned to the two groups of A and B in a 1:1 ratio. Finally, all the 120 subjects were evaluated in two groups of intervention and placebo (Figure 1).

The mean age of patients was 52.34±8.95 years, and 115 (95.8%) of them were males. There was no significant difference between the two groups in terms of age and sex. All surgeries in this study were lower extremity and pelvic surgeries.

As shown in Table 1, the difference in systolic blood pressure before induction of anesthesia (after albuterol spray administration) between the group that received albuterol spray and the group that did not receive it was statistically significant (P=0.04). The mean difference of mean arterial blood pressure before anesthesia induction (after administration of albuterol spray) and before tracheal extubation (after re-administration of albuterol spray) between the two groups were statistically significant. Also, the difference in mean of SPO2 before tracheal extubation (after re-administration of albuterol spray) between the two groups were statistically significant (P=0.03).

**Table 1:** Hemodynamic indices in two groups of patients

	Hemodynamic indices	Groups	Mean±SD	P-value
**Before induction of** **anesthesia (after** **administration of** **albuterol spray)**	Heart rate	A (Albuterol spray)	80.66±14.36	0.24
		B (Placebo)	80.76±14.93	
	Systolic BP	A (Albuterol spray)	120.50±16.94	0.04
		B (Placebo)	110.41±12.89	
	Diastolic BP	A (Albuterol spray)	72.33±11.51	0.49
		B (Placebo)	72.58±11.02	
	MAP	A (Albuterol spray)	85.88±12.61	0.02
		B (Placebo)	87.25±12.09	
	SPO2	A (Albuterol spray)	98.43±1.26	0.63
		B (Placebo)	98.71±1.34	
**Before tracheal extubation** **(after re-administration of** **albuterol spray)**	Heart rate	A (Albuterol spray)	93.76±18.15	0.30
		B (Placebo)	94.36±19.18	
	Systolic BP	A (Albuterol spray)	134.16±19.74	0.24
		B (Placebo)	136.58±18.92	
	Diastolic BP	A (Albuterol spray)	73.11±9.44	0.13
		B (Placebo)	72.91±8.92	
	MAP	A (Albuterol spray)	90.10±11.61	0.04
		B (Placebo)	91.72±10.91	
	SPO2	A (Albuterol spray)	99.41±1.63	0.03
		B (Placebo)	97.41±1.55	

As shown in Table 2, respiratory wheezing and frequent coughing before anesthesia induction (after albuterol spray administration) were less in the intervention group (receiving albuterol spray) than the control group. However in terms of other symptoms such as bronchospasm, nausea, vomiting, dyspnea and shivering, there was no significant difference between the two groups (P>0.05).

**Table 2:** Frequency of adverse events in two groups before anesthesia induction (after albuterol spray administration)

Adverse events		Group A (Albuterol spray) N (%)	Group B (Placebo) N (%)	P-value
Wheezing	Yes	5 (8.3)	18 (30)	0.003
	No	55 (91.7)	42 (70)	
Bronchospasms	Yes	0 (0)	0 (0)	1
	No	60 (100)	60 (100)	
Recurrent coughing	Yes	0 (0)	4 (6.7)	0.04
	No	60 (100)	56 (93.3)	
Dyspnea	Yes	1 (1.7)	2 (3.3)	0.55
	No	59 (98.3)	58 (96.7)	
Nausea	Yes	0 (0)	0 (0)	1
	No	60 (100)	60 (100)	
Shivering	Yes	0 (0)	0 (0)	1
	No	60 (100)	60 (100)	

According to the results of the chi-square test before induction of anesthesia, out of 60 patients who received albuterol spray, 5 patients (8.3%) had wheezing, but in the control group, 18 patients (30%) had wheezing (p=0.003). Also, before induction of anesthesia, none of the 60 patients who received albuterol spray had frequent cough, but in the control group, 4 patients (6.7%) had frequent cough (p=0.04). As shown in Table 3, wheezing and bronchospasm before tracheal extubation (after re-administration of albuterol spray) were lower in the intervention group (receiving albuterol spray) than in the control group. However, other symptoms such as frequent cough, nausea, dyspnea, and shivering were not statistically significantly different between the two groups (p>0.05). Before tracheal extubation, out of 60 patients who received albuterol spray, 16 (26.7%) had wheezing, but in the control group, 25 patients (41.7%) had wheezing (P = 0.04). Also, before tracheal extubation, none of the 60 patients who received albuterol spray had bronchospasm, but in the control group, 5 patients (8.3%) had bronchospasm (P=0.01). No significant side effects were detected in the albuterol-treated group.

**Table 3:** Frequency of adverse events in two groups of patients before tracheal extubation (after represcribing albuterol spray)

Adverse events		Group A (Albuterol spray) N(%)	Group B (Placebo) N(%)	P-value
Wheezing	Yes	16 (26.7)	25 (41.7)	0.04
	No	44 (73.3)	35 (58.3)	
Bronchospasms	Yes	0 (0)	5 (8.3)	0.01
	No	60 (100)	55 (91.7)	
Recurrent coughing	Yes	13 (21.7)	17 (28.3)	0.39
	No	47 (78.3)	43 (71.7)	
Dyspnea	Yes	8 (13.3)	4 (6.7)	0.22
	No	52 (86.7)	56 (93.3)	
Nausea	Yes	38 (63.3)	44 (73.3)	0.23
	No	22 (36.7)	16 (26.7)	
Shivering	Yes	51 (85)	44 (73.3)	0.11
	No	9 (15)	16 (26.7)	

## Discussion

The results of the present study showed that wheezing and frequent coughing before induction of anesthesia (after administration of albuterol spray) in the intervention group (receiving albuterol spray) were less in the intervention group than the control group. Also, wheezing and bronchospasm before tracheal extubation (after re-administration of albuterol spray) were less in the intervention group than in the control group.

A study by Etamadi et al. showed that out of 30 heavy smoking patients during general anesthesia who received salbutamol spray, 21 had no problems, and only 9 patients from this group had bronchospasm or wheezing or irritating cough or decreased oxygen saturation. Also, out of 30 patients in the control group, 13 were without complications while 17 patients of this group had one or more complications such as bronchospasm, wheezing, irritating cough, and depressed blood oxygen saturation, which were resolved with the necessary medical measures. None of the patients had acute respiratory distress syndrome after surgery (11).

In the study of Imantalab et al. on 225 heavy smoking patients who had a history of smoking (20 packs/year) with normal spirometry and without any respiratory symptoms, who underwent coronary artery bypass surgery, patients were divided into 3 groups (75 in each group).

In the first group were the smoking patients who had not been treated and had smoked before the surgery. The second group consisted of smoking patients who were treated with 3 drugs, Salbutamol-Atronet-Beclomethasone, inhaled 2 puffs twice a day, 10 to 14 days before the surgery, and they had smoked before the operation. In the third group were smokers who had quit smoking for 8 weeks. In the first, second and third groups, out of 75 patients, 35, 23 and 31 suffered from at least one postoperative complications. The frequency of coughing and wheezing and the average duration of mechanical ventilation and the incidence of reoperation due to repeated coughing in the group that was treated with salbutamol-atronate-beclomethasone were statistically lower than the other two groups (13).

Smoking is a major trigger of chronic mucus hyper-secretion, decreases the protective host responses to infection, impairs the capability of cilia to clear mucus and causes air flow obstruction. Also, due to the occurrence of hypoxia in these patients, pulmonary vascular resistance and blood viscosity increase (14,15).

Ninety percent of smoking patients have chronic arterial insufficiency. Although patients are advised for cessation of smoking 8 to 12 weeks before elective surgery or at least 18 hours before surgery, it is observed that these patients continue their smoking habit. The harmful substances in cigarette smoke are not fully known. Some of these substances produce bronchitis in the trachea and large bronchi, and others cause emphysema in smaller bronchioles and alveoli (16–19). In these patients, when chronic airway obstruction occurs, lung damage is irreversible and smoking is the leading cause of COPD. In the pre-operative visit of smoking and COPD patients, the severity of the disease and the possibility of bronchospasm and infection should be evaluated (6,19).

Albuterol is a β2 agonist whose possible mechanisms of action include relaxation of bronchial smooth muscles, stimulation of mucociliary clearance, stabilization of mesenteries, reduction of edema caused by increased vascular permeability, reduction of pulmonary artery pressure, improvement of diaphragm contraction and inhibition of transmission of parasympathetic ganglia in the airways. Beta-agonists have antitussive effects, due to the drug's effect on endothelial and epithelial permeability, inhibiting the release of mediators, and stimulating mucociliary clearance (20,21).

The results of a study by Kim, Eun et al., who investigated the effects of albuterol administration before intubation on the emergence coughing are inconsistent with the present study. In that study, the researchers concluded that emergence coughing is very common, and smoking has no effect on cough during emergence from anesthesia. Also, using albuterol has no beneficial effect on emergence coughing (22). One possible explanation for this inconsistency may be using albuterol spray before intubation and extubation in our study, compared to using albuterol only before intubation in Euns Kim et al's study.

Also, in the study of Hurwitz et al. it was shown that smoking reduced the function of pulmonary vessels and airways, so that albuterol consumption did not cause a significant decrease in pulmonary vascular resistance in both smoking and non-smoking groups. However on the other hand, it caused a greater decrease in airway vascular resistance in non-smokers than in smokers (23). The results of another study by Ramgolam et al. showed that premedication with salbutamol to children aged between 6 and 16 years and at high risk of perioperative respiratory, adverse events including bronchospasm, laryngospasm, airway obstruction, desaturation, coughing and stridor, did not reduce their risk of these adverse events (24). However, the results of another study confirmed the efficacy of albuterol premedication administration before tonsillectomy under general anesthesia in young children in reduction of rates of perioperative respiratory adverse events compared to the rates in children who received placebo (25).

There were some limitations to our study. We did not assess some potential confounders in this study such as preoperative anxiety. Moreover, determining the exact dosage of albuterol that reached the bronchus under the administration of metered-dose inhaler was difficult. In addition, the absence of preoperative pulmonary function data was another limitation of this study.

In conclusion, according to the results of the preset study, it seems that using one puff of albuterol spray (2.5 micrograms) before induction of anesthesia can significantly reduce wheezing and frequent coughing in COPD patients under general anesthesia. Also, re-administration of one puff of albuterol spray (2.5 micrograms) before tracheal extubation can significantly reduce wheezing and bronchospasm compared to the control group. In other words, the preventive use of albuterol spray will be useful in reducing the incidence of bronchospasm and hypoxia, and frequent coughing after anesthesia in COPD patients who smoke.
